# Evaluating phecodes, clinical classification software, and ICD-9-CM codes for phenome-wide association studies in the electronic health record

**DOI:** 10.1371/journal.pone.0175508

**Published:** 2017-07-07

**Authors:** Wei-Qi Wei, Lisa A. Bastarache, Robert J. Carroll, Joy E. Marlo, Travis J. Osterman, Eric R. Gamazon, Nancy J. Cox, Dan M. Roden, Joshua C. Denny

**Affiliations:** 1 Departments of Biomedical Informatics, Vanderbilt University Medical Center, Nashville, TN, United States of America; 2 Departments of Medicine, Vanderbilt University Medical Center, Nashville, TN, United States of America; 3 Vanderbilt Genetic Institute and the Division of Genetic Medicine, Vanderbilt University, Nashville, TN, United States of America; 4 Department of Clinical Epidemiology, Academic Medical Center, University of Amsterdam, Amsterdam, Netherlands; 5 Department of Biostatistics and Bioinformatics, Academic Medical Center, University of Amsterdam, Amsterdam, Netherlands; 6 Department of Department of Psychiatry, Academic Medical Center, University of Amsterdam, Amsterdam, Netherlands; 7 Department of Clinical Pharmacology, Vanderbilt University Medical Center, Nashville, TN, United States of America; University of Chicago, UNITED STATES

## Abstract

**Objective:**

To compare three groupings of Electronic Health Record (EHR) billing codes for their ability to represent clinically meaningful phenotypes and to replicate known genetic associations. The three tested coding systems were the International Classification of Diseases, Ninth Revision, Clinical Modification (ICD-9-CM) codes, the Agency for Healthcare Research and Quality Clinical Classification Software for ICD-9-CM (CCS), and manually curated “phecodes” designed to facilitate phenome-wide association studies (PheWAS) in EHRs.

**Methods and materials:**

We selected 100 disease phenotypes and compared the ability of each coding system to accurately represent them without performing additional groupings. The 100 phenotypes included 25 randomly-chosen clinical phenotypes pursued in prior genome-wide association studies (GWAS) and another 75 common disease phenotypes mentioned across free-text problem lists from 189,289 individuals. We then evaluated the performance of each coding system to replicate known associations for 440 SNP-phenotype pairs.

**Results:**

Out of the 100 tested clinical phenotypes, phecodes exactly matched 83, compared to 53 for ICD-9-CM and 32 for CCS. ICD-9-CM codes were typically too detailed (requiring custom groupings) while CCS codes were often not granular enough. Among 440 tested known SNP-phenotype associations, use of phecodes replicated 153 SNP-phenotype pairs compared to 143 for ICD-9-CM and 139 for CCS. Phecodes also generally produced stronger odds ratios and lower p-values for known associations than ICD-9-CM and CCS. Finally, evaluation of several SNPs via PheWAS identified novel potential signals, some seen in only using the phecode approach. Among them, rs7318369 in *PEPD* was associated with gastrointestinal hemorrhage.

**Conclusion:**

Our results suggest that the phecode groupings better align with clinical diseases mentioned in clinical practice or for genomic studies. ICD-9-CM, CCS, and phecode groupings all worked for PheWAS-type studies, though the phecode groupings produced superior results.

## Introduction

The near ubiquity of electronic health records (EHRs) represents an unprecedented opportunity to leverage large-scale healthcare data for discovery.[1, 2] Compared with randomized controlled trials or traditional observational cohorts, EHR-based studies offer several distinct advantages, including cost efficiency[3, 4], scale[5, 6], and the ability to conduct longitudinal analyses[7–9]. These advantages make EHRs a viable and efficient model for clinical and genomic research[10], including the potential to analyze hundreds of human diseases, drug responses, and many observable clinical traits. EHRs have proven particularly useful for phenome-wide association studies (PheWAS); however, currently, there is no EHR-derived “reference phenome” available for such research.[10] Most PheWAS, and indeed many other EHR studies, leverage International Classification of Diseases, Ninth Revision, Clinical Modification (ICD-9-CM) billing codes to define phenotypes. In this study, we evaluated three different ICD-based coding systems to enable clinical and genomic research.

The World Health Organization (WHO) established and maintained the original version of International Classification of Diseases, Ninth Revision (ICD-9) to track morbidity and mortality statistics across the world. The United States Department of Health & Human Services and the Centers for Medicare and Medicaid Services (CMS) further created ICD-9-CM as an extension of ICD-9 primarily for billing purposes. ICD-9-CM codes have been widely used as a record of patient diagnoses in clinical practice and health management for decades. ICD-9-CM uses three (e.g. 250 “diabetes mellitus”) to five digits (e.g. 250.00 “type 2 diabetes mellitus without mention of complication”) to describe diseases and syndromes. The 2015 edition of ICD-9-CM has 22,401 distinct codes. Many of these codes are not billable codes (e.g., diabetes mellitus requires a 5-digit specification, so code “250” is not an allowable code). These codes are arranged hierarchically into nineteen large chapters (e.g., “Diseases of the digestive system”), 160 sections (e.g., “Noninfectious enteritis and colitis”), and 1,247 3-digit categories (e.g., 250.* “diabetes mellitus”). The first three digits describe the general condition of a patient and therefore have been commonly used to represent disease categories.

Despite their convenience, using ICD-9-CM for phenotypic analyses remains challenging because not all codes are organized meaningfully for the purpose of high-throughput phenotypic analyses. ICD-9-CM separates active diagnosis of many diseases from a history of the same disease into different chapters, e.g. malignant neoplasm of breast (174.9) and personal history of malignant neoplasm of breast (V10.3). In addition, other diseases are separated for other reasons: sleep apnea has codes in the “Diseases of the nervous system” chapter (327.2) and in the “Symptoms, signs, and ill-defined conditions” (780.57). In other cases, logical numerical groupings combine diseases that are genetically or pathophysiologically distinct: 250.* aggregates both type 1 and type 2 diabetes mellitus. ICD-10 solves the last example but not the former ones. Thus, simply using the natural, numerical ICD groupings can introduce inaccuracy when phenotyping diseases.

To facilitate clinical research using ICD codes, the Agency for Healthcare Research and Quality introduced the Clinical Classification Software for ICD-9-CM-CM (CCS) in 1999.[11] CCS reorganizes disparate ICD-9-CM codes into a smaller number of clinically meaningful categories. These categories are more useful for presenting descriptive statistics than individual ICD-9-CM codes. Similar to three-digit ICD-9-CM codes, CCS is an adequate solution for research that requires disease representations at a gross level. Nevertheless, with only 294 mutually exclusive categories (850 leaf diagnoses) in its 2015 version, CCS may lack sufficient granularity for many clinical studies.

In addition, most clinical studies using the EHR require not only a phenotype definition for cases (those *with* the disease phenotype) but also one for controls (those *without* the disease phenotype and related conditions that might be the phenotype of interest).[12–15] For example, studies of type 2 diabetes often exclude subjects with any other type of diabetes.[16] Neither ICD-9-CM nor CCS provides a ready-made approach to automatically exclude populations of patients that are “possible” cases or have similar or potentially overlapping disease states (e.g., type 1 diabetes and secondary diabetes mellitus). In prior analyses, researchers often use 3-digit ICD-9-CM codes (parent codes) for exclusions, which could miss codes in other parts of diagnosis system (e.g., gestational diabetes, abnormal glucose, and secondary diabetes all have different 3-digit codes than type 1 and type 2 diabetes mellitus). The absence of such codes can result in a contamination of cases in the control population and decrease statistical power.

Knowledgeable clinical researchers conducting focused studies on a particular disease often address these issues with custom approaches; however, our goal was to compare multiple schemas for phenotyping in high-throughput manner, such as PheWAS. In the process of developing the PheWAS approach within EHRs, we developed an aggregation schema for ICD-9-CM codes that attempted to represent distinct diseases and traits for primary clinical or genetic research while also pairing with groupings to identify reasonable control groups.[17, 18] These phenotype codes, or “phecodes”, aggregate one or more related ICD-9-CM codes into distinct diseases or traits. We manually reviewed all ICD-9-CM codes (including useful E and V codes) to reorganize them into phecodes, boosted by clinical co-occurrence data to identify like phenotypes in disparate sections of the ICD-9-CM code system (for example the ICD-9-CM code group 162 representing lung cancers and the code group V10.1 representing history of lung cancers) and existing phenotypes used in prior GWAS studies. Phecodes were first introduced in 2010 with 733 distinct phenotype codes.[17] We have continuously updated the phecode groupings with the additional clinical experts helping with revisions of different domains, such as cardiology and oncology. The latest version of the phecodes involves 1,866 hierarchical phenotype codes and groups 15,558 ICD-9-CM codes.[18]

Phecodes are arranged hierarchically, like CCS. The phecode hierarchical structure includes disease codes not present in the ICD-9-CM billing hierarchy, such as “inflammatory bowel disease” as the parent phenotype for “Crohn's disease” and “ulcerative colitis.” Like other clinical terminologies, a leaf code refers to one that has no children while a top code refers to one that has no parent phecode.

To facilitate a high-throughput phenomic analysis with a quality control population, we also provide an algorithm-driven method to identify control populations for each phecode. We predefined a relevant control group for each phecode and individuals with related diseases do not serve as controls for that phecode (e.g., an individual with either type 1 diabetes, secondary diabetes mellitus, or elevated blood glucose codes cannot serve as a control for an analysis of type 2 diabetes.[17, 18]

Phecodes have been successfully used in a number of PheWAS to replicate hundreds of known genetic associations and discover new ones, some with subsequent validation studies.[17–23] Other EHR-based PheWAS studies have used ungrouped ICD-9-CM codes.[24–26] One study has identified variable results using phecodes and raw or custom ICD-9-CM codes with five cardiovascular phenotypes.[27] However, the applicability of these coding systems for PheWAS has not been rigorously compared to other groupings of ICD-9-CM codes. Each of these groupings (ICD-9-CM, CCS, and phecodes) is freely available. In this paper, we compared ICD-9-CM, CCS, and our recently updated phecode mappings for their ability to represent common diseases. We then tested each system’s ability to replicate known genetic associations as an orthogonal evaluation of their phenotype mapping accuracy.

## Materials and methods

We employed two distinct tests to compare the three coding systems for high-throughput phenotypic analyses using EHRs. The first test was to compare the capability of each coding systems to accurately represent clinical phenotypes using clinical records and phenotypes pursued in genome-wide association studies (GWAS). Second, to demonstrate the feasibility of using each code system for high-throughput phenomic analysis, we performed PheWAS with ICD-9-CM codes, CCS, and phecodes using genetic variants with known associations.

### Evaluating accuracy of phenotype representation

We tested the ability of each coding system to accurately represent phenotypes derived from two sources: the National Human Genome Research Institute (NHGRI) Catalog of Published GWAS[28] and clinical records of 189,289 individuals taken from Vanderbilt University Medical Center’s EHR.

We randomly selected 25 phenotypes from the GWAS Catalog that 1) were atomic phenotypes (i.e., compound phenotypes, such as *bipolar disorder and schizophrenia*, were ignored), 2) had at least 25 individuals with the phenotype in the EHRs (as identified by Systematized Nomenclature of Medicine—Clinical Terms [SNOMED CT] codes–see below), and 3) were clinical phenotypes likely to be in a billing code system (e.g., we ignored phenotypes like “hair color”).

Another 75 clinical diseases (exclude 25 selected diseases from GWAS catalog) were selected from the most commonly documented diseases in free-text problem lists taken from 189,289 individuals taken from Vanderbilt University Medical Center’s EHR. We selected the “Significant Medical Problems and Diagnoses” section of the provider-maintained “Patient Summary”, which is corresponds roughly to an amalgamation of the “past medical history” and “active problems” and is used across both inpatient and outpatient care. These problems can be coded to SNOMED CT but are also often free text. These were mapped to SNOMED CT terms using the KnowledgeMap concept identifier[29], ignoring negated terms[30]. From these, we selected the 75 most common problems (as represented in SNOMED CT) to attempt to manually map to disease phenotypes (described below). These 75 phenotypes from problem lists and the 25 from GWAS catalog were mutually exclusive.

Two authors (WQW and LB) independently reviewed these diseases, located the most appropriate ICD-9-CM code, CCS code, and phecode representing each of these disease phenotypes. For ICD-9-CM, billable or non-billable terms could be selected (e.g., “250.*” is not billable but could be chosen to represent all of type 1 and type 2 diabetes). The two authors also determined whether or not the chosen code was an *exact* or *inexact* match to the phenotype. Since our goal was the evaluation of the accuracy of phenotype representation, only single codes could be selected–*ad hoc* creation of custom code groupings was not allowed. An *exact* match shows an equivalent semantic representation (e.g., “ICD-9-CM code 708.*” → “*Urticaria***”**). An *inexact* one suggests the best-matched representation is either broader (less specific than the target disease, e.g. “ICD-9-CM code 278.* [*Overweight*, *obesity and other hyperalimentation*]*” → “obesity”)* or narrower (more specific than the target disease, e.g. “CCS code 3.2 [*diabetes mellitus without complication*]” → “*diabetes mellitus*”). Both broader and narrower matches were classified as *inexact*.

Cohen’s kappa was calculated to estimate inter-rater agreement between the two reviewers. Another author (JCD) adjudicated all labeling conflicts. For each coding system, we defined the phenotype accuracy score as the number of exactly matched phenotypes divided by the number of total phenotypes reviewed. Phenotype accuracy performance (phecode vs. ICD-9-CM and phecode vs. CCS) were compared using McNemar's test.

### Evaluating ability to replicate known phenotype-genotype associations

#### Population

To compare the utility of the three coding systems for PheWAS, we leveraged existing genetic data in BioVU, Vanderbilt’s DNA biobank linked to de-identified EHR data.[31] We compared the ability of each to replicate known genotype-phenotype associations in a PheWAS for variants with known associations. We used the common variants with known phenotype associations in the GWAS Catalog that were also present on the Illumina HumanExome genotyping array, which is enriched by inclusion of GWAS catalog SNPs. We used 35,842 BioVU individuals with genotyping on this array and EHR records. We used a minor allele frequency (MAF) cutoff of 1%. The 35,842 individuals were genotyped for previous studies but in general were selected for individuals with longitudinal care at Vanderbilt.[31] We considered all available SNPs included in the current GWAS Catalog updated to the April 17, 2015 version. We considered all phenotypes that matched or nearly matched with NHGRI Catalog phenotypes.

#### Case definitions

For each phenotype, we queried all ICD-9-CM codes from each individual’s EHR based on the best-matched representation of each coding systems. For example, to define cases with 174 (Malignant neoplasm of female breast) for ICD-9-CM, we selected all individuals who had any child codes under 174 –e.g., individuals with 174.0 (Malignant neoplasm of nipple and areola of female breast) or 174.9 (Malignant neoplasm of breast [female], unspecified) were qualified. However, if an individual had the ICD-9-CM code V10.3 (Personal history of malignant neoplasm of breast) but no 174.* codes, the individual was only qualified as a case of breast cancer for both phecode and CCS schemes, since both of those code systems group these codes together under the “breast cancer” phenotype.

#### Control definitions

We used the built-in exclusion definitions to define controls for phecode, as described briefly above and in previous work.[17, 18] Because CCS and ICD-9-CM do not have embedded control definitions, we used the parent code to define a range of codes to exclude. For example, to define controls for a case with 244.0 (postsurgical hypothyroidism), we selected all individuals who did not have any codes under the parent code 244 (acquired hypothyroidism)–e.g., individuals with any 244.1(other postablative hypothyroidism), 244.9 (unspecified acquired hypothyroidism) could not serve as controls. Similarly, the CCS code 4.1.1 (Acute posthemorrhagic anemia) has an exclude range of 4.1 (Anemia). In the breast cancer example above, since these V10.3 and 174.* are not co-located in ICD-9-CM, they exist as two separate phenotypes in a PheWAS analysis using ICD-9-CM codes. Moreover, individuals with V10.3 end up serving as a control for individuals with 174.* codes when using ICD-9-CM codes. Both CCS and phecode would prevent such individuals from serving as controls.

#### PheWAS

Following alignment of phecodes to GWAS Catalog phenotypes, we performed a PheWAS analysis using each coding system to replicate known genetic associations. We chose three SNPs (rs35391, rs731839 and rs769449) to study that had strong replication of known phenotypes in the above study in at least one of the methods but had differences in the replication phenotypes studied. Among available SNPs to test with replications, this selection was essentially a convenience selection chosen to highlight some of the differences in encoding.

Tested phenotype-genotype associations were restricted to those identified in our prior analysis, for which we manually curated all associations in the GWAS catalog for direction of effect, ancestry, and sex tested. Tested genotypes were then restricted to those common variants available on the exome chip platform. We only tested associations in which there were at least 25 individuals with the phenotype in the population. Since these were previously known associations, we used a nominal p value of 0.05 as our threshold to define replication. We also evaluated the number of associations that cross a Bonferroni threshold (p = 0.05/440 = 1.14×10^−4^). All association tests used logistic regression assuming an additive model and were adjusted for age, gender, and sex. Since most of our exome samples were of European ancestry, only European ancestry samples were used in this analysis. The European ancestry was determined by principal component analysis.

All statistical analyses were performed in R, version 3.2.5 (https://www.r-project.org/). PheWAS and plotting used the R PheWAS package. ^32^

## Results

A general comparison of the three coding system was listed in Table 1. ICD-9-CM has the largest number of leaf (22,401) and top (1,247) codes while CCS has the least number of codes (leaf: 850; top: 294). The reviewed results of the 100 randomly selected diseases and their mappings are in the S1 Table. The kappa score between the two reviewers was 0.94 (95% confidence interval: 0.89–1.00), suggesting excellent agreement.

**Table 1:** Comparison of the evaluated coding schema.

	ICD-9_CM	AHRQ CCS	Phecodes
Availability	n/a	https://www.hcup-us.ahrq.gov/toolssoftware/ccs/ccs.jsp	http://phewascatalog.org
Number of Phenotypes	L: 22,401; T:1,247^*^	L: 850; T: 294	L: 1,360; T: 659^**^
Embedded control Definition	No	No	Yes
Control definition	All individuals except those with relevant top codes	All individuals except those with parent CCS code	All individuals except those with exclude codes in PheWAS control definition
Example control exclusion for “Atrial fibrillation” (ICD-9-CM 427.31, CCS 7.2.9.3, phecode 427.21)	427.^*^	7.2.9	426–427.99

L: leaf codes; T: top codes

* “top codes” for ICD-9-CM are 3 digit ICD-9-CM codes

** 153 codes are both top and leaf codes.

Overall, the phenotype accuracy score for phecodes (83/100) was significantly higher than the phenotype accuracy score for ICD-9-CM (53/100; p<0.01) and CCS (32/100; p<0.01). Of the 25 most common problem list diagnoses, 22 (88%) had exactly matching phecodes, while only 16 (64%) and 7 (28%) had exact matches in the ICD-9-CM and CCS code systems, respectively. Of the 75 diseases from the GWAS catalog, 81% (61) had exactly matching phecodes, while 49% (37) and 33% (25) had exactly matching ICD-9-CM or CCS codes, respectively. The ICD-9-CM codes were generally too narrow for a target phenotype while CCS were too broad. For example, the closest ICD-9-CM code for hypertension was 401 (essential hypertension), which excluded codes like 402.* (Hypertensive heart disease) and 405.* (Secondary hypertension). The best CCS code for hyperlipidemia was 3.6 (disorders of lipid metabolism), which included code like 272.1 (pure hyperglyceridemia) and 272.3 (hyperchylomicronemia).

**Fig 1:**
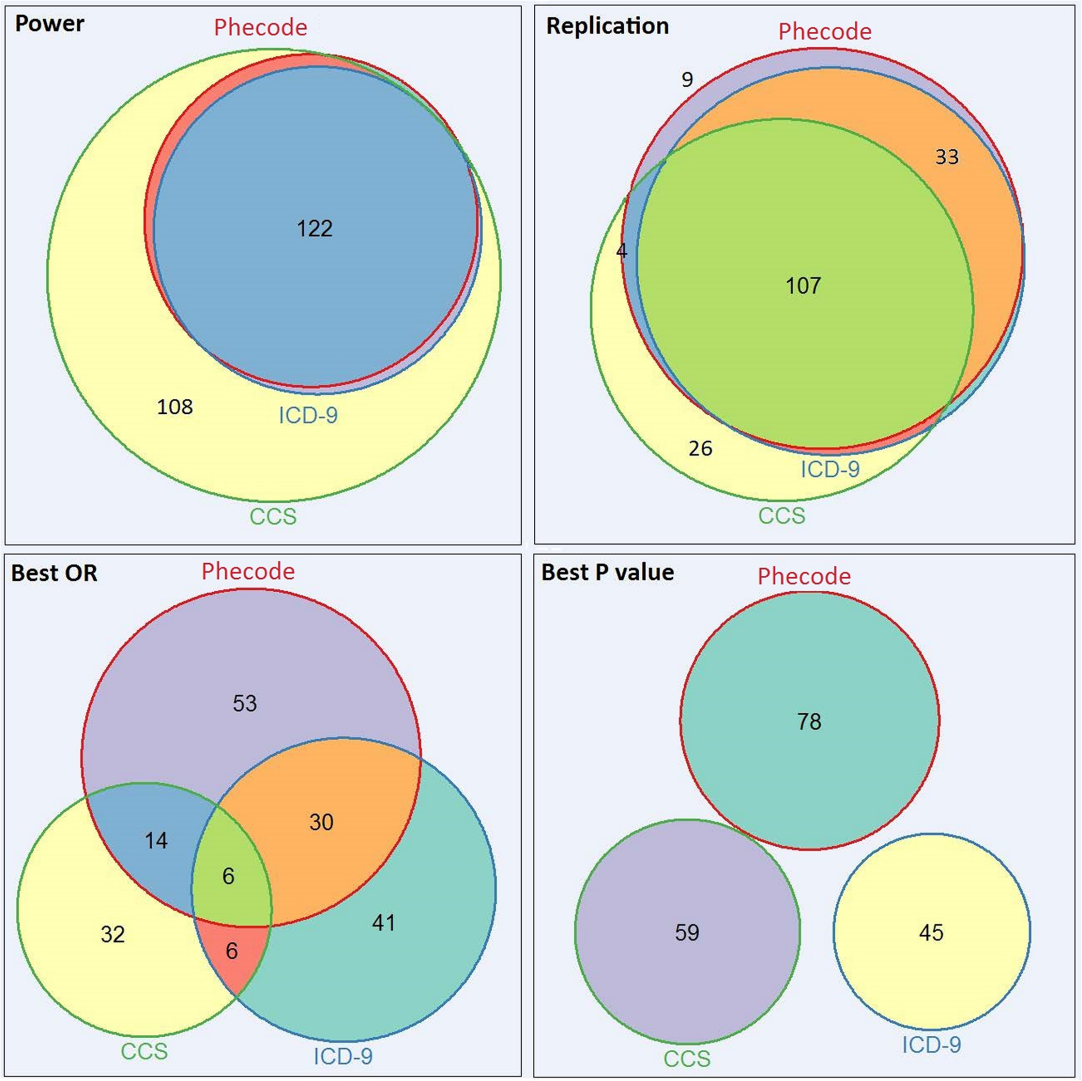
Weighted Venn diagrams of the distributions of power-enabled tests, replicated associations, best ORs, and best P values with CCS, ICD-9-CM, and phecodes. Each color represents a resource.

In the SNP-phenotype replication analysis, we identified 440 known SNP-phenotype associations (including 400 unique SNPs and 100 distinct phenotypes) available for testing. CCS typically identified more cases than ICD-9-CM and phecodes (Fig 1, upper left); however, this was often a result of a lower level of phenotype granularity in CCS that grouped more codes together than desired (e.g. 3.6 [disorders of lipid metabolism] for hyperlipidemia). Using phecodes, we successfully replicated 153 (34.8%) associations at p<0.05. Using ICD-9-CM, we replicated 143 (32.5%). Using CCS, we replicated 139 (31.6%) associations in spite of the apparent increase in statistical power over ICD-9-CM and phecodes due to the increased sample size (Table 2). However, many of the CCS phenotypes with large sample sizes were broader than the target phenotypes (e.g. study of ulcerative colitis using CCS 9.6.2, which represents both regional enteritis and ulcerative colitis).

**Table 2:** Results of tests for genetic replications on 440 SNP and phenotype pairs.

	Phecode	ICD-9-CM	CCS
Replication (P<0.05)	153 (34.8%)	143 (32.5%)	139 (31.6%)
Replication (Bonferroni)	43 (9.8%)	34 (7.7%)	34 (7.7%)
Best OR	103	83	58
Best P Value	78	45	59

We then compared the number of known associations we would have found if using a stricter, “discovery” type approach that only considered as significant those associations exceeding a Bonferroni threshold. Among these replications, 43 of 153 PheWAS replications have been found as significant using this threshold compared to 34 using either ICD-9-CM or CCS. The results of McNemar's exact tests suggested significant differences between the replication rates of phecodes and the other two systems (p<0.05). In addition, our results suggest that phecodes typically resulted in the strongest odd ratios (103 in total, Fig 1, lower left) and P values (78 in total, Fig 1, lower right).

**Fig 2:**
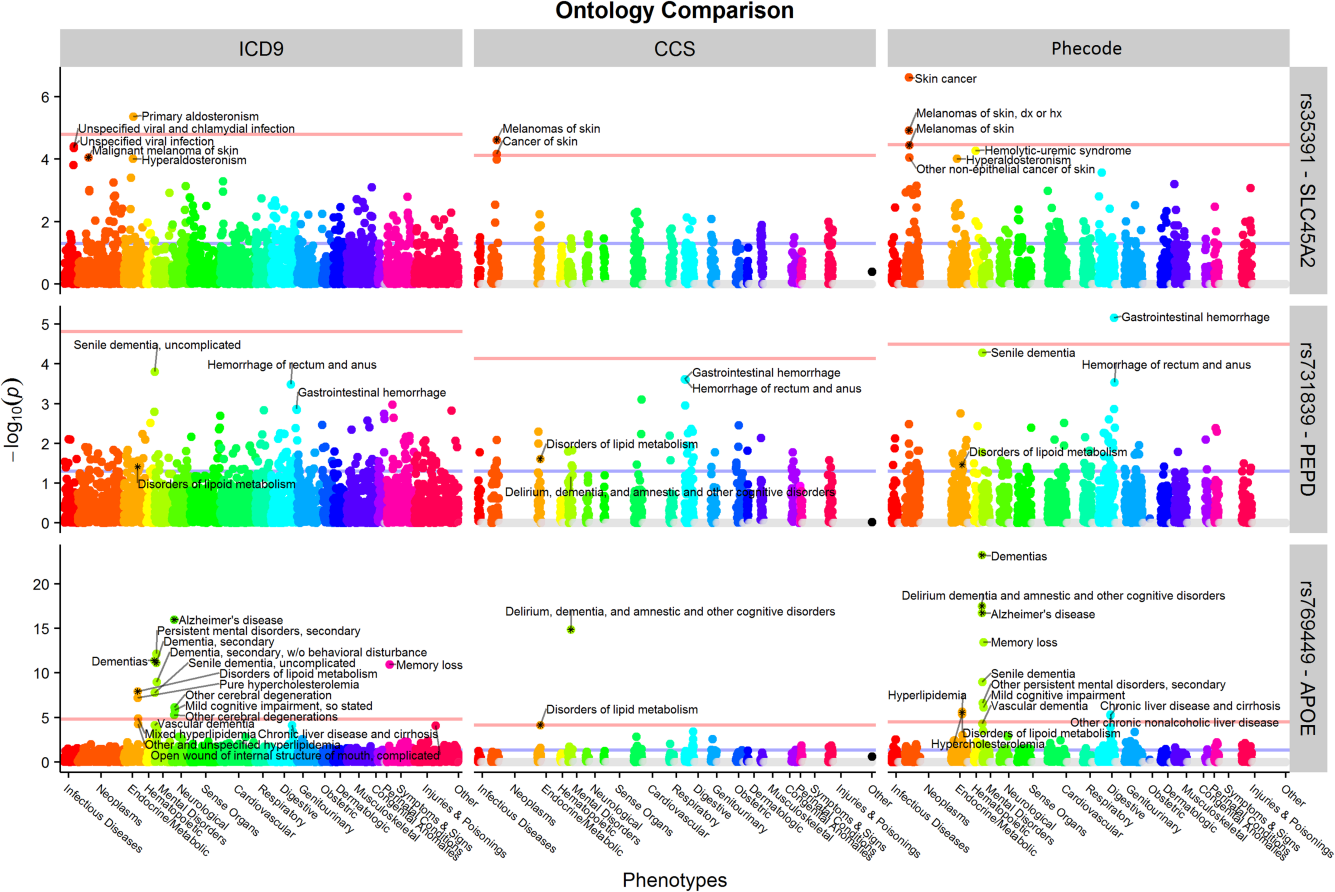
PheWAS results of three SNPs (rs35391, rs731839 and rs769449) showed that phecodes outperformed ICD-9-CM and CCS.

**Fig 3:**
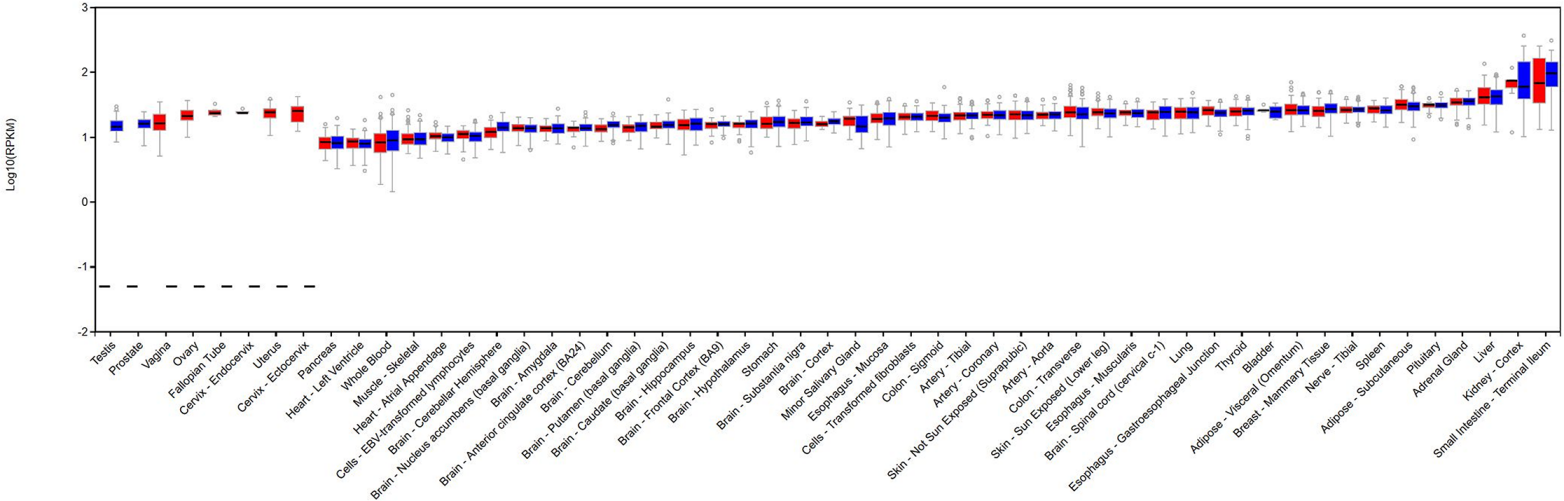
PEPD expression results suggest strong association with the gastrointestinal tract.

**Fig 4:**
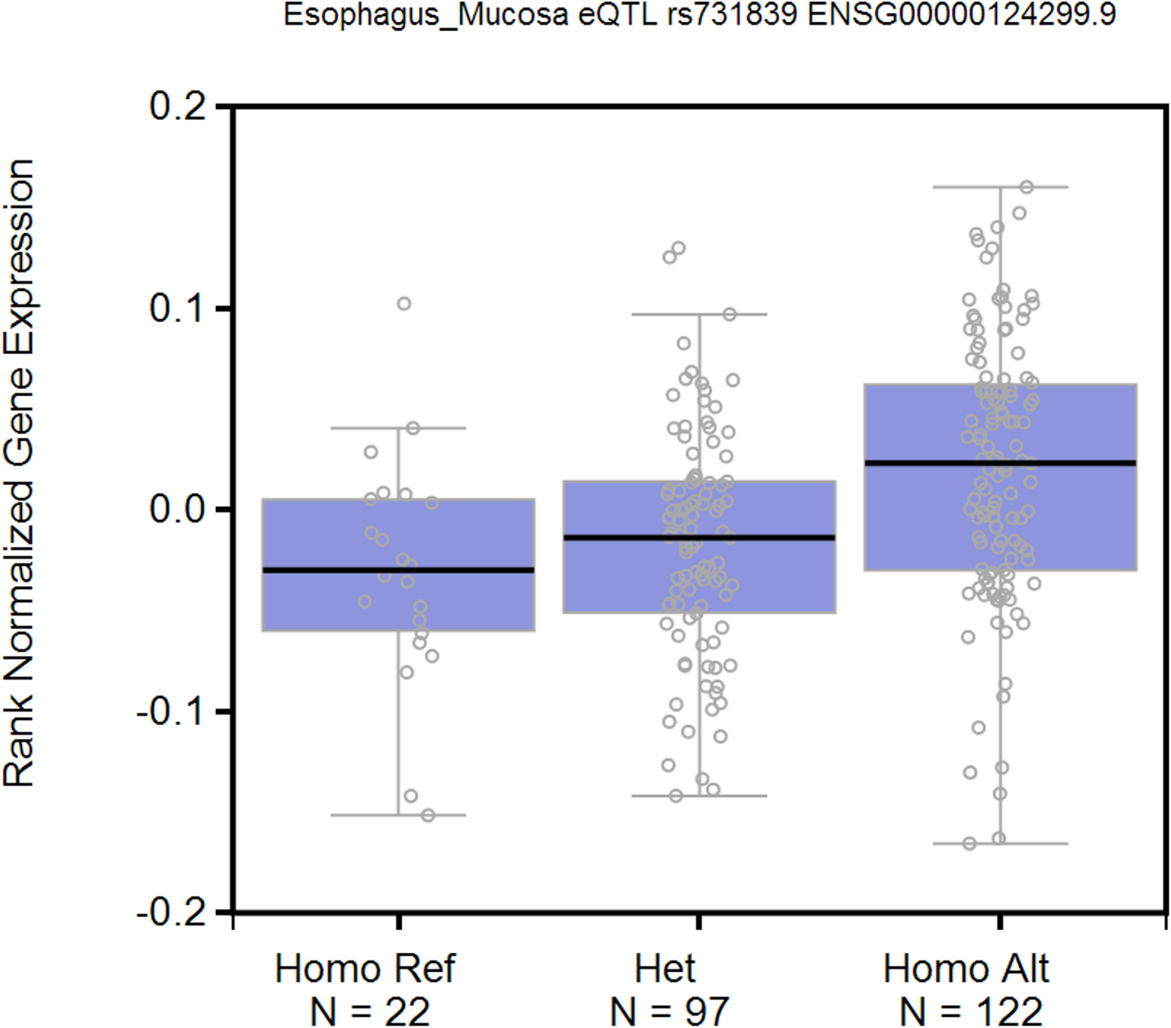
SNP rs731839 is a cis acting eQTL for PEPD in esophagus mucosa.

Finally, we picked three SNPs (rs35391, rs731839 and rs769449) to demonstrate the benefits of using phecodes for PheWAS analyses (Fig 2). rs35391 (*SLC45A2*) is known to be associated with the risk of skin cancer.[32, 33] We replicated this finding using CCS and phecodes, but not ICD-9-CM codes. Phecodes offered the most significant P value across the three code systems. Similarly, use of phecodes replicated more known phenotypes for rs769449 (*APOE*). In addition, neither CCS nor ICD-9-CM found any significant associations for rs731839 (*PEPD*) while phecodes provided a potential association with gastrointestinal hemorrhage (p = 6.93E-6). Evidence from GTEx suggests that this common polymorphism may result in a tissue-specific decrease in expression of *PEPD* in the esophageal mucosa (p = 2.7E-6, Figs 3 and 4).[34, 35] Lower levels of *PEPD* may disrupt the integrity of the gut mucosa by limiting synthesis of collagen, and lead to bleeding. The known association of rs731839 is with lipid levels, which was observed significantly associated with disorders of lipid metabolism using all of the three system.[36]

## Discussion

An obvious gap impeding efficient use of EHR data for large-scale phenomic analyses is the lacking of rapid, accurate, and meaningful phenotypic characterization. We evaluated three coding systems for organizing billing codes for their meaningfulness and ability to characterize the clinical phenome. Our results suggest that phecodes provide groupings of diseases codes that more closely align with phenotypes found on natural-language EHR problem lists and those previously studied in GWAS. As expected, we found that all three coding systems demonstrate utility for phenome-wide studies. However, we found that using phecodes replicated more known genetic associations than using ICD-9-CM codes or CCS. Our results also point to a generalized approach for use of other billing code grouping systems for validating phenotype groupings.

Phecodes better represented disease phenotypes among the three coding systems for the 100 phenotypes manually mapped. Eighty-three percent of tested diseases were exactly matched to a phecode compared to 53% and 32% for ICD-9-CM and CCS, respectively. The higher relevance of phecodes was consistent across common diseases based on EHRs (81%, 61/75) and randomly selected ones from the GWAS catalog (88%, 22/25). In comparison, ICD-9-CM better matched the GWAS catalog phenotypes (16/25, 64%) than the EHR common diseases (37/75, 49%). Many common disease phenotypes could not be exactly matched to a high-level categorical ICD-9-CM code because relevant ICD-9-CM codes were stored in distant branches, e.g. specific cancer codes (140–239) and their relevant “personal history of” cancer codes (V10). Combining codes is not trivial across diverse diseases. In our data set, 5.7% (16,114 vs. 283,870) of individuals with at least one V10.* code (personal history of cancers) did not have a diagnosis code for cancer (140–239).

Clearly, custom groupings can be made in the ICD-9-CM for any one given disease, but our purposes were to evaluate a broad use case without requiring the creation of novel aggregations. CCS and phecode both provide these aggregations, grouping related but scattered codes into meaningful diseases, such as cancers and differentiating types 1 and 2 of diabetes. However, in many cases, CCS lacked the granularity to capture a particular disease concept, such as regional enteritis or ulcerative colitis. Only 32% of diseases could be exactly matched to a CCS code. For example, the CCS code 3.1.2 (*Other thyroid disorders*), although a leaf code, encapsulates numerous diverse diseases and symptoms like Graves’ disease, hypothyroidism, thyroiditis, and thyroid cysts. Phecodes offered more detailed phenotypes than CCS but less detailed phenotypes than ICD-9-CM. Grouping like codes together (personal history of a cancer and the diagnosis of that cancer) also decreases the contamination of cases in the controls. The reorganization can increase the sample size of a study while reducing the number of independent phenotypes tested, thereby improving statistical power.

In this study, aggregating billing codes into meaningful phenotypes yielded better replication rates and typically better p values. Leader et al. compared five gold standard phenotypes to ICD-9-CM 5-digit and 3-digit diseases and phecodes. They reported similar results for several phenotypes and differing results for more detailed cardiovascular phenotypes in their studies. They found phecodes were less granular than their desired phenotypes.[27] In our study, phecodes replicated more known GWAS catalog associations than ICD-9-CM and CCS using either a nominal replication p-value or a Bonferroni correction. We observed that phecodes successfully facilitated replicating known genetic associations with various cancers while ICD-9-CM and CCS did not, e.g. rs11249433 with breast cancer and rs801114 with basal cell carcinoma (S2 Table). Phecodes also typically yielded better odds ratios (103 of 153 replicated genotype-phenotype pairs) and lower p-values (78 of 153 replicated genotype-phenotype pairs).

Importantly, we did not observe any associations exclusively replicated using ICD-9-CM codes, which might be expected given their greater granularity. However, we observed ten associations that were replicated only using CCS (see S2 Table). CCS often included a larger number of other relevant diseases than the other systems, increasing the sample size. For example, rs3197999 is known to be associated with both ulcerative colitis and Crohn's disease. Phecodes and ICD-9-CM codes have a more accurate representation of either of the two diseases than CCS because the most specific code of CCS (9.6.2, Regional enteritis and ulcerative colitis) combined both phenotypes. However, we could identify only 200 cases by using phecodes or ICD-9-CM and neither one replicated the association between rs3197999 and ulcerative colitis nor rs3197999 and Crohn's disease. CCS, by including codes of both two diseases, identified 448 cases and successfully replicated the two associations, albeit only as a single unit (Of note, phecodes also include a higher-level “inflammatory bowel disease” phenotype which also replicated this association, but was not the association tested in this analysis since it was not the GWAS Catalog phenotype.) In addition, our study suggested the potential of using phecodes to discover novel associations. We discovered a potential novel association between a polymorphism of *PEPD* and gastrointestinal hemorrhage.

Both CCS and phecodes are freely available and can easily be applied to EHR data (https://www.hcup-us.ahrq.gov/toolssoftware/ccs/ccs.jsp; http://phewascatalog.org/). This study, along with our previous findings[18, 37], demonstrated the fine granularity and usability of phecodes. To be useful, such coding systems need to be periodically updated. An R package to facilitate translating ICD-9-CM to phecodes and conducting phenotypic analyses is freely available.[38]

Several limitations regarding the creation and evaluation of phecodes should be mentioned. First, phecodes currently only aggregate ICD-9-CM codes. In fact, much effort has been made to cross mapping current ICD-9-CM codes to ICD-10, and efforts are underway to map and validate an ICD-10 translation to phecode.[39] Future phecodes should include both, although the bulk of extant billing codes available for research are ICD-9-CM in the US. Ultimately, the generation of phecodes incorporating both ICD-9-CM and ICD-10 should be informed by actual clinical practice and billing patterns. Billing code phenotypes (regardless of the system used) cannot cover phenotypes that are not relevant to a diagnosis (e.g., eye color), longitudinal phenotypes (e.g., progression of a disease), or most drug responses (e.g., clopidogrel failure[40] or cough on angiotensin converting enzyme inhibitors[41]). Use of other resources, such as laboratory or textual EHR data, Human Phenotype Ontology (HPO)[42], participant provided data, or physical exams, remains important to obtain a comprehensive representation of phenotypes. Active integration of HPO into the Unified Medical Language System may facilitate use of HPO for EHR-based research.[43]

It should also be noted that a number of other notable efforts of the representation of disease phenotypes have been pursued and represent opportunities for future incorporation into the EHR. They include HPO and Disease Ontology (DO).[44] HPO embraces phenotypic abnormalities encountered in the medical literature, Orphanet[45], DECIPHER[46], and Online Mendelian Inheritance in Man (OMIM).[47] DO has been developed collaboratively across several institutions and aims at providing an extensive cross mapping of disease concepts among Medical Subject Headings (MeSH), ICD-9-CM, ICD-10, National Cancer Institute Thesaurus (NCIt), SNOMED CT, and OMIM. Both ontologies formally describe disease phenotype concepts and provide semantic relationships among them. Nevertheless, neither HPO nor DO focuses on the linkage between its concepts and their corresponding diagnosis codes currently used in clinical practice at this time. Some DO concepts have cross mappings to ICD-9-CM codes while many concepts do not, including, for example, both types of diabetes mellitus and pneumonia. Such a lack of connection between their phenotype concepts and formal representation of clinical data significantly impedes their broad usage for clinical data. Moreover, concerns about the specificity and accuracy of billed diagnoses in the ICD-9-CM (or in clinical problem lists, for that matter)[48, 49] could make such automated mappings between ICD-9-CM and HPO or DO potentially problematic in the real-world use case. Future efforts, such as that of the Global Alliance for Genes and Health, are pursuing efforts to incorporate more detailed, structured phenotype encoding into EHRs, and could provide new dimensions of EHR-based phenomic investigation.

## Supporting information

S1 Table Results of manual code matching to GWAS and common EHR phenotypes. (DOCX)

S2 Table Example phenotype-SNP associations that can be replicated by only one coding scheme. (DOCX)
