# Supplementary material for: Evaluating phecodes, clinical classification software, and ICD-9-CM codes for phenome-wide association studies in the electronic health record
Source: PLoS One. 2017 Jul 7;12(7):e0175508. doi: 10.1371/journal.pone.0175508 (PMC5501393; doi:10.1371/journal.pone.0175508)
Supplement: S1 Table — (DOCX) [file pone.0175508.s001.docx]

S1 Table. Results of manual code matching to GWAS and common EHR phenotypes.

|  | **Phenotype** | **ICD-9-CM** | | | **phecode** | | | **ASHQ CCS** | | | **Comments** |  |
| --- | --- | --- | --- | --- | --- | --- | --- | --- | --- | --- | --- | --- |
|  |  | code | description | M^*^ | code | description | M | code | description | M |  |  |
| **25 diseases from GWAS Catalog** | Alzheimer's disease | 331 | Alzheimer's disease | 1 | 290.1 | Alzheimer's disease | 1 | 5.4 | dementia, amnestic & other cognitive disorders | 0 |  |  |
|  | HIV disease | 042 | HIV disease | 0 | 71 | HIV disease | 1 | 1.3.1 | HIV infection | 1 | ICD-9-CM has no asymptomatic HIV codes while phecode & CCS do |  |
|  | Narcolepsy | 347 | Cataplexy & narcolepsy | 1 | 347 | Cataplexy & narcolepsy | 1 | 6.9.2 | Other CNS disorders | 0 |  |  |
|  | Hepatitis B | 70.3 | Hep B w/t hepatic coma | 0 | 70.2 | Viral Hep B | 1 | 1.3.2 | Hep | 0 |  |  |
|  | Pancreatic Ca | 157 | Pancreatic Ca | 1 | 157 | Pancreatic Ca | 1 | 2.2.4 | Pancreatic Ca | 1 |  |  |
|  | Lung Ca | 162 | Ca of trachea, bronchus, lung | 0 | 165.1 | Ca of bronchus, lung | 1 | 2.3 | Ca of bronchus, lung | 1 | ICD-9-CM does not include history codes |  |
|  | Hypothyroidism | 244.9 | Hypothyroidism NOS | 1 | 244.4 | Hypothyroidism NOS | 1 | 3.1.2 | Other thyroid disorders | 0 |  |  |
|  | Dupuytren's disease | 728.6 | Dupuytren's disease | 1 | 728.7 | Dupuytren's disease | 1 | 13.8 | Other connective tissue disease | 0 |  |  |
|  | Obesity | 278 | Overweight & obesity | 0 | 278.1 | Obesity | 1 | 3.11.2 | Obesity | 0 | PheWAS excludes overweight |  |
|  | Glaucoma | 365 | Glaucoma | 1 | 365 | Glaucoma | 1 | 6.7.3 | Glaucoma | 1 |  |  |
|  | Urinary bladder Ca | 188 | Ca of bladder | 0 | 189.2 | Ca of bladder | 1 | 2.9.1 | Ca of bladder | 1 | ICD-9-CM has no history codes |  |
|  | Drinking behavior | 303 | Alcohol dependence | 0 | 317 | Alcohol-related disorders | 0 | 5.11 | Alcohol-related disorders | 0 | no exact codes for all |  |
|  | POAG | 365.1 | POAG | 1 | 365.1 | POAG | 1 | 6.7.3 | Glaucoma | 0 |  |  |
|  | AF & AFL | 427.3 | AF & AFL | 1 | 427.2 | AF & AFL | 1 | 7.2.9 | Cardiac dysrhythmias | 0 |  |  |
|  | Malaria | 84 | Malaria | 1 | 133 | Arboviral diseases | 0 | 1.4 | Other infections; incl. parasitic | 0 |  |  |
|  | MD | 296 | Episodic MD | 0 | 296 | MD | 1 | 5.8 | MD | 1 |  |  |
|  | PCOS | 256.4 | PCOS | 1 | 256.4 | PCOS | 1 | 3.4 | Other endocrine disorders | 0 |  |  |
|  | Endometrial Ca | 182.0 | Ca of corpus uteri, exc. isthmus | 1 | 182 | Ca of uterus | 0 | 2.6.1 | Ca of uterus | 0 |  |  |
|  | Vitiligo | 709 | Vitiligo | 1 | 694.1 | Vitiligo | 1 | 12.4 | Other skin disorders | 0 |  |  |
|  | Celiac disease | 579 | Celiac disease | 1 | 557.1 | Celiac disease | 1 | 9.12.3 | gastrointestinal disorders, NOS | 0 |  |  |
|  | Keloid | 701.4 | Keloid scar | 1 | 701.4 | Keloid scar | 1 | 12.4 | Other skin disorders | 0 |  |  |
|  | BD | 296.8 | Other & NOS BD | 0 | 296.1 | BD | 1 | 5.8.1 | BD | 1 |  |  |
|  | UC | 556 | UC | 1 | 555.2 | UC | 1 | 9.6.2 | Regional enteritis & UC | 0 |  |  |
|  | Uterine fibroids | 218 | Uterine leiomyoma | 1 | 218.1 | Uterine leiomyoma | 1 | 2.16.1 | Benign neoplasm of uterus | 0 |  |  |
|  | Colorectal Ca | 153 | colon Ca | 0 | 153 | Colorectal Ca | 1 | 2.1.1 | Ca of colon | 0 |  |  |
| **75 commonly documented diseases from problem lists** | HTN | 401.9 | essential HTN, NOS | 0 | 401 | HTN | 1 | 7.1 | HTN | 1 |  |  |
|  | HLD | 272.4 | HLD, NOS | 0 | 272.1 | HLD | 1 | 3.6 | Disorders of lipid metabolism | 0 |  |  |
|  | GERD | 530.8 | GERD | 1 | 530.1 | GERD | 1 | 9.4.1.2 | Other esophageal disorders | 0 |  |  |
|  | CHD | 429.2 | CHD, NOS | 0 | 411 | Ischemic Heart Disease | 0 | 7.2 | Diseases of the heart | 0 | no exact codes for all |  |
|  | AAA | 441 | Aortic aneurysm & dissection | 0 | 442.11 | AAA | 1 | 7.4.2.1 | AAA w/t rupture | 0 |  |  |
|  | T2DM | 250 | T2DM, NOS | 0 | 250.2 | T2DM | 1 | 3.2 | DM w/t complication | 0 |  |  |
|  | pneumonia | 486 | Pneumonia, NOS | 0 | 480 | Pneumonia | 1 | 8.1.1 | Pneumonia | 1 |  |  |
|  | HCL | 272 | Pure HCL | 1 | 272.1 | HCL | 1 | 3.6 | Disorders of lipid metabolism | 0 |  |  |
|  | OA | 715 | OA & allied disorders | 1 | 740 | OA | 1 | 13.2.2 | OA | 1 |  |  |
|  | Epilepsy | 345 | Epilepsy & recurrent seizures | 0 | 345.1 | Epilepsy | 1 | 6.4.1 | Epilepsy | 1 |  |  |
|  | Osteoporosis | 733 | Osteoporosis | 1 | 743.1 | Osteoporosis | 1 | 13.4 | Osteoporosis | 1 |  |  |
|  | Asthma | 493 | Asthma | 1 | 495 | Asthma | 1 | 8.3 | Asthma | 1 |  |  |
|  | Allergic rhinitis | 477 | Allergic rhinitis | 1 | 476 | Allergic rhinitis | 1 | 8.9 | Other upper respiratory disease | 0 |  |  |
|  | Anemia | 285.9 | Anemia NOS | 0 | 280 | Iron deficiency anemias | 0 | 4.1.3 | Deficiency & other anemia | 1 |  |  |
|  | COPD | 496 | COPD, NOS | 0 | 496 | COPD | 1 | 8.2 | COPD & bronchiectasis | 1 |  |  |
|  | Shingles | 53 | Shingles | 1 | 53 | Shingles | 1 | 1.3.3.1 | Shingles | 1 |  |  |
|  | CVA | 434.9 | CVA, NOS w/ cerebral infarction | 0 | 433.2 | CVA w/ cerebral infarction | 1 | 7.3.1.2 | Occlusion of cerebral arteries | 0 |  |  |
|  | Kidney Stones | 592 | Calculus of kidney | 1 | 594.1 | Calculus of kidney | 1 | 10.1.5.1 | Calculus of kidney | 1 |  |  |
|  | Basal cell Ca | 173 | Other skin Ca | 0 | 172.2 | Basal cell Ca | 1 | 2.4.2 | Other non-epithelial Ca of skin | 0 |  |  |
|  | AMI | 410.9 | AMI of unspecified site | 0 | 411.2 | AMI | 1 | 7.2.3 | AMI | 1 |  |  |
|  | RA | 714.0 | RA | 1 | 714.1 | RA | 1 | 13.2.1 | RA & related disease | 0 |  |  |
|  | Migraine Disorder | 346.9 | Migraine NOS | 0 | 340 | Migraine | 1 | 6.5.1 | Migraine | 1 |  |  |
|  | Prostate cancer | 185 | Prostate Ca | 0 | 185 | Prostate Ca | 1 | 2.8.1 | Prostate Ca | 1 | ICD-9-CM includes no history codes |  |
|  | CHF | 428 | CHF | 1 | 428.1 | CHF | 1 | 7.2.11.1 | CHF | 1 |  |  |
|  | Diverticulosis | 562 | Diverticula of intestine | 0 | 562.1 | Diverticulosis | 1 | 9.6.4.1 | Diverticulosis | 1 |  |  |
|  | Sleep apnea | 780.57 | Unspecified sleep apnea | 0 | 327.3 | Sleep apnea | 1 | 18 | unclassified; all E codes | 0 |  |  |
|  | Urticaria | 708 | Urticaria | 1 | 947 | Urticaria | 1 | 17.1.9 | Allergic reactions | 0 |  |  |
|  | Chronic sinusitis | 473 | Chronic sinusitis | 1 | 475 | Chronic sinusitis | 1 | 8.1.5.2 | Chronic sinusitis | 1 | ICD-9-CM does not include 784.91 |  |
|  | Dysphagia | 787.2 | Dysphagia | 1 | 532 | Dysphagia | 1 | 9.12.2 | Dysphagia | 1 |  |  |
|  | PE | 415.1 | PE & infarction | 0 | 415.1 | PE & infarction | 0 | 7.2.6 | Pulmonary heart disease | 0 |  |  |
|  | Urinary incontinence | 788.3 | Urinary incontinence | 1 | 599.4 | Urinary incontinence | 1 | 10.1.8.3 | Other & unspecified genitourinary symptoms | 0 |  |  |
|  | Renal failure | 586 | Renal failure unspecified | 0 | 585.2 | Renal failure unspecified | 0 | 10.1.2 | Acute & unspecified renal failure | 1 | AHRQ includes acute renal failure |  |
|  | Cataract | 366 | Cataract | 0 | 366 | Cataract | 1 | 6.7.1 | Cataract | 0 | Phecode includes history code |  |
|  | Psoriasis | 696 | Psoriasis & similar disorders | 0 | 696.4 | Psoriasis | 0 | 12.2 | Other inflammatory condition of skin | 0 | Phecode includes 696.1 |  |
|  | Spinal stenosis | 724.0 | Spinal stenosis, excl. cervical | 0 | 720 | Spinal stenosis | 1 | 13.3.3.2 | Spinal stenosis; lumbar region | 0 |  |  |
|  | IBS | 564.1 | IBS | 1 | 564.1 | IBS | 1 | 9.12.3 | Other & unspecified gastrointestinal disorders | 0 |  |  |
|  | Osteomyelitis | 730 | Osteomyelitis, periostitis, & other infections involving bone | 0 | 710.1 | Osteomyelitis | 1 | 13.1 | Infective arthritis & osteomyelitis (except that caused by TB or STD) | 0 |  |  |
|  | Retinopathy | 362 | Other retinal disorders | 0 | 362 | Other retinal disorders | 0 | 6.7.2.3 | Other retinal disorders | 0 |  |  |
|  | Nail Fungus | 110.1 | Dermatophytosis of nail | 1 | 110.11 | Dermatophytosis of nail | 1 | 1.2.2 | Other mycoses | 0 |  |  |
|  | Chronic pain | 338.2 | Chronic pain | 1 | 338.2 | Chronic pain | 1 | 6.9.3 | Other nervous system symptoms & disorders | 0 |  |  |
|  | End stage renal disease | 585.6 | End stage renal disease | 1 | 585.32 | End stage renal disease | 1 | 10.1.3 | Chronic renal failure | 0 |  |  |
|  | UTI | 599.0 | UTI, site not specified | 0 | 591 | UTI | 1 | 10.1.4.3 | UTI; site not specified | 0 | Phecode includes V13.12 |  |
|  | Varicose veins | 454 | Varicose veins of lower extremities | 0 | 454* | Varicose veins | 1 | 7.5.2 | Varicose veins of lower extremity | 0 |  |  |
|  | Hemorrhoids | 455 | Hemorrhoids | 1 | 455 | Hemorrhoids | 1 | 7.5.3 | Hemorrhoids | 1 |  |  |
|  | Dehydration | 276.51 | Dehydration | 1 | 276.5 | Hypovolemia | 0 | 3.8.2 | Hypovolemia | 0 |  |  |
|  | Hydronephrosis | 591 | Hydronephrosis | 1 | 595 | Hydronephrosis | 1 | 10.1.6.1 | Hydronephrosis | 1 |  |  |
|  | Peptic ulcer | 533 | Peptic ulcer, site unspecified (excl. gastic & duodenal ulcers) | 0 | 531 | Peptic ulcer (excl. esophageal ulcer) | 0 | 9.4.2 | Gastroduodenal ulcer (except hemorrhage) | 0 | Phecode is the most complete, but excludes esophageal ulcers |  |
|  | Mixed hyperlipidemia | 272.2 | Mixed hyperlipidemia | 1 | 272.13 | Mixed hyperlipidemia | 1 | 3.6 | Disorders of lipid metabolism | 0 |  |  |
|  | Vitamin D deficiency | 268 | Vitamin D deficiency | 1 | 261.4 | Vitamin D deficiency | 1 | 3.5.2 | Other malnutrition | 0 |  |  |
|  | Gastroparesis | 536.3 | Gastroparesis | 1 | 536.3 | Gastroparesis | 1 | 9.4.4 | Other disorders of stomach & duodenum | 0 |  |  |
|  | Hypercalcemia | 275.42 | Hypercalcemia | 1 | 275.6 | Hypercalcemia | 1 | 3.11.1 | Disorders of mineral metabolism | 0 |  |  |
|  | Hypoglycemia | 251.2 | Hypoglycemia NOS | 0 | 251.1 | Hypoglycemia | 1 | 3.4 | Other endocrine disorders | 0 | Phecode includes 251.0 & 251.1 |  |
|  | Bacteremia | 790.7 | Bacteremia | 1 | 038.3 | Bacteremia | 1 | 1.1.2.6 | Unspecified septicemia | 1 |  |  |
|  | Fluid overload | 276.6 | Fluid overload | 1 | 276.6 | Fluid overload | 0 | 3.8.5 | Other fluid & electrolyte disorders | 0 | ICD-9-CM includes transfusion associated circulatory overload |  |
|  | Gall Stone | 574 | Cholelithiasis | 0 | 574 | Cholelithiasis & cholecystitis | 1 | 9.7 | Biliary tract disease | 0 |  |  |
|  | Left ventricular hypertrophy | 429.3 | Cardiomegaly | 0 | 416 | Cardiomegaly | 0 | 7.2.7 | Other & ill-defined heart disease | 0 |  |  |
|  | Intestinal obstruction | 560 | Intestinal obstruction w/t mention of hernia | 1 | 560 | Intestinal obstruction w/t mention of hernia | 1 | 9.6.3 | Intestinal obstruction w/t hernia | 1 |  |  |
|  | Meningitis | 322 | Meningitis of unspecified cause | 0 | 320 | Meningitis | 1 | 6.1.1 | Meningitis (except that caused by TB or STD) | 1 | Phecode & AHRQ include infectious Meningitis |  |
|  | Nephrolithiasis | 592.0 | Calculus of kidney | 1 | 594.1 | Calculus of kidney | 1 | 10.1.5.1 | Calculus of kidney | 1 |  |  |
|  | Meniere's disease | 386.0 | Meniere's disease | 1 | 386.1 | Meniere's disease | 1 | 6.8.2 | Conditions associated with dizziness or vertigo | 0 |  |  |
|  | Deep vein thrombosis | 453.4 | Venous embolism & thrombosis of deep vessels of lower extremity | 0 | 452.2 | Deep vein thrombosis | 1 | 7.5.1.2 | Other venous embolism & thrombosis | 0 |  |  |
|  | Diastolic heart failure | 428.3 | Diastolic heart failure | 1 | 428.4 | Diastolic heart failure | 1 | 7.2.11 | Congestive heart failure; nonhypertensive | 0 |  |  |
|  | Crohn's disease | 555 | Regional enteritis | 1 | 555.1 | Regional enteritis | 1 | 9.6.2 | Regional enteritis & ulcerative colitis | 0 |  |  |
|  | RLS | 333.94 | RLS | 1 | 327.71 | RLS | 1 | 6.2.3.2 | Other unspecified hereditary & degenerative nervous conditions | 0 |  |  |
|  | Gout | 274 | Gout | 1 | 274.1 | Gout | 1 | 3.7 | Gout & crystal arthropathies | 0 |  |  |
|  | T1DM | 250 | T2DM or T1DM | 0 | 250.1 | T1DM | 1 | 3.2 | DM w/t complication | 0 |  |  |
|  | Osteopenia | 733.90 | Disorder of bone & cartilage, unspecified | 0 | 743.9 | Osteopenia or disorder of bone & cartilage | 0 | 13.9 | Other bone disease & musculoskeletal deformities | 0 |  |  |
|  | Diverticulitis | 562 | Diverticula of intestine | 0 | 562.2 | Diverticulitis | 1 | 9.6.4.2 | Diverticulitis | 1 |  |  |
|  | Lymphedema | 457.1 | Other lymphedema | 0 | 450 | Noninfectious disorders of lymphatic channels | 0 | 7.5.4 | Other diseases of veins & lymphatics | 0 |  |  |
|  | Degenerative disorder of macula | 362.5 | Degeneration of macula & posterior pole of retina | 1 | 362.2 | Degeneration of macula & posterior pole of retina | 1 | 6.7.2.3 | Other retinal disorders | 0 |  |  |
|  | Systemic sclerosis | 710.1 | Systemic sclerosis | 0 | 709.3 | Systemic sclerosis | 1 | 13 | Diseases of the musculoskeletal system & connective tissue | 0 | Phecode includes 517.2 |  |
|  | Pancreatitis | 577.1 | Chronic pancreatitis | 0 | 577.2 | Chronic pancreatitis | 0 | 9.9.2 | Chronic pancreatitis | 0 | None includes acute pancreatitis |  |
|  | Insomnia | 327.0* | Organic disorders of initiating & maintaining sleep | 0 | 327.4 | Insomnia | 1 | 6.9.2 | Other central nervous system disorders | 0 |  |  |
|  | Raynaud's disease | 443.0 | Raynaud's syndrome | 1 | 443.1 | Raynaud's syndrome | 1 | 7.4.4.2 | Other unspecified circulatory disease | 0 |  |  |
|  | Diplopia | 368.2 | Diplopia | 1 | 368.2 | Diplopia & disorders of binocular vision | 0 | 6.7.4 | Blindness & vision defects | 0 |  |  |
| Total | |  | 53 | |  | 83^**^ | |  | 32 | |  |  |
|  | ^*^ Match type (1 represents an *exact match* while 0 represents an *inexact match*)  ^**^Significantly higher than the other two coding schemes, P<0.01  AAA: abdominal aortic aneurysm  AF : atrial fibrillation  AFL: atrial flutter  AMI: acute myocardial infarction  BD: bipolar disorder  Ca: cancer  CHD: coronary heart disease  CHF: congestive heart failure  CNS: central nervous system  COPD: chronic obstructive pulmonary disease  CVA: cerebrovascular accident  DM: diabetes mellitus  GERD: gastresophageal reflux disease  HCL: hypercholesterolemia  Hep: hepatitis  HIV: human immunodeficiency virus  HLD: hyperlipidemia  HTN: hypertension  IBS: irritable bowel syndrome  MD: mood disorders  NOS: not otherwise specified or unspecified  OA: osteoarthritis  PCOS: polycystic ovary syndrome  PE: Pulmonary embolism  POAG: primary open-angle glaucoma  RA: rheumatoid arthritis  RLS: restless legs syndrome  UC: ulcerative colitis  UTI: urinary tract infection T2DM: type 2 diabetes mellitus | | | | | | | | | | | |
