# Supplementary material for: Evaluating phecodes, clinical classification software, and ICD-9-CM codes for phenome-wide association studies in the electronic health record
Source: PLoS One. 2017 Jul 7;12(7):e0175508. doi: 10.1371/journal.pone.0175508 (PMC5501393; doi:10.1371/journal.pone.0175508)
Supplement: S2 Table — (DOCX) [file pone.0175508.s002.docx]

S2 Table. Example phenotype-SNP associations that can be replicated by one coding scheme.

| **SNP** | **Phenotype** | **Phecode** | | **ICD-9-CM** | | **CCS** | | **Replicated** | **P** |
| --- | --- | --- | --- | --- | --- | --- | --- | --- | --- |
|  |  | **code** | **cases** | **code** | **cases** | **code** | **cases** |  |  |
| rs4977574 | Myocardial infarction | 411.2 | 1,601 | 410 | 960 | 7.2.3 | 960 | PheWAS | 1.80E-04 |
| rs6010620 | Glioma | 191.11 | 501 | 191 | 464 | 2.11.4 | 547 | PheWAS | 1.40E-02 |
| rs11249433 | Breast cancer | 174.11 | 1,438 | 174 | 1,240 | 2.5 | 1,491 | PheWAS | 1.60E-02 |
| rs1746048 | Myocardial infarction | 411.2 | 1,601 | 410 | 960 | 7.2.3 | 960 | PheWAS | 1.70E-02 |
| rs4430796 | Endometrial cancer | 182 | 304 | 182 | 257 | 2.6.1 | 303 | PheWAS | 2.30E-02 |
| rs4939827 | Colorectal cancer | 153 | 1,025 | 153 | 734 | 2.1 | 1,024 | PheWAS | 2.70E-02 |
| rs944289 | Thyroid cancer | 193 | 532 | 193 | 494 | 2.11.5 | 532 | PheWAS | 3.00E-02 |
| rs801114 | Basal cell carcinoma | 172.21 | 324 | 173 | 1,841 | 2.4.2 | 2,346 | PheWAS | 3.20E-02 |
| rs20541 | Psoriasis | 696.4 | 378 | 696 | 390 | 12.2 | 1,703 | PheWAS | 4.80E-02 |
| rs3130573 | Systemic sclerosis | 709.3 | 196 | 710.1 | 194 | 13.7 | 882 | CCS | 6.70E-04 |
| rs1024161 | Alopecia areata | 704.11 | 29 | 704 | 29 | 12.4 | 7,325 | CCS | 1.10E-03 |
| rs3764650 | Alzheimer's disease | 290.11 | 243 | 331 | 243 | 5.4 | 1,283 | CCS | 1.90E-02 |
| rs6499188 | Ulcerative colitis | 555.2 | 194 | 556 | 194 | 9.6.2 | 448 | CCS | 3.10E-02 |
| rs3197999 | Ulcerative colitis | 555.2 | 194 | 556 | 194 | 9.6.2 | 448 | CCS | 3.10E-02 |
| rs3197999 | Crohn's disease | 555.1 | 251 | 555 | 251 | 9.6.2 | 448 | CCS | 3.10E-02 |
| rs254560 | Ulcerative colitis | 555.2 | 194 | 556 | 194 | 9.6.2 | 448 | CCS | 3.20E-02 |
| rs11465804 | Crohn's disease | 555.1 | 251 | 555 | 251 | 9.6.2 | 448 | CCS | 3.30E-02 |
| rs3213094 | Psoriasis vulgaris | 696.41 | 299 | 696.1 | 299 | 12.2 | 1,703 | CCS | 4.10E-02 |
| rs7804356 | Type 1 diabetes | 250.1 | 375 | 250 | 353 | 3.2 | 5,536 | CCS | 3.20E-02 |
